# Markers of Restenosis after Percutaneous Transluminal Balloon Angioplasty in Patients with Critical Limb Ischemia

**DOI:** 10.3390/ijms24109096

**Published:** 2023-05-22

**Authors:** Elvira V. Sobolevskaya, Oleg A. Shumkov, Mikhail A. Smagin, Andrey E. Guskov, Alexandra V. Malysheva, Victor V. Atuchin, Vadim V. Nimaev

**Affiliations:** 1 Laboratory of Surgical Lymphology and Lymph-Detoxication, Research Institute of Clinical and Experimental Lymphology—Branch of the Institute of Cytology and Genetics, SB RAS, Novosibirsk 630117, Russia; 2 Laboratory of Scientometrics and Scientific Communications, Russian Research Institute of Economics, Politics and Law in Science and Technology, Moscow 127254, Russia; 3 Laboratory of Optical Materials and Structures, Institute of Semiconductor Physics, SB RAS, Novosibirsk 630090, Russia; 4 Research and Development Department, Kemerovo State University, Kemerovo 650000, Russia; 5 Department of Industrial Machinery Design, Novosibirsk State Technical University, Novosibirsk 630073, Russia; 6 R&D Center “Advanced Electronic Technologies”, Tomsk State University, Tomsk 634034, Russia

**Keywords:** atherosclerosis, markers, restenosis, percutaneous transluminal angioplasty, peripheral arterial disease, critical limb ischemia

## Abstract

Among cardiovascular diseases, chronic obliterating lesions of the arteries of lower extremities, which are one of the important problems of modern healthcare, are distinguished. In most cases, the cause of damage to the arteries of lower extremities is atherosclerosis. The most severe form is chronic ischemia, characterized by pain at rest and ischemic ulcers, ultimately increasing the risk of limb loss and cardiovascular mortality. Therefore, patients with critical limb ischemia need limb revascularization. Percutaneous transluminal balloon angioplasty is one of the least invasive and safe approaches, with advantages for patients with comorbidities. However, after this procedure, restenosis is still possible. Early detection of changes in the composition of some molecules as markers of restenosis will help screen patients at the risk of restenosis, as well as find ways to apply efforts for further directions of inhibition of this process. The purpose of this review is to provide the most important and up-to-date information on the mechanisms of restenosis development, as well as possible predictors of their occurrence. The information collected in this publication may be useful in predicting outcomes after surgical treatment and will also find new ways for the target implication to the mechanisms of development of restenosis and atherosclerosis.

## 1. Introduction

### 1.1. The Collection of Materials for the Review

The collection of materials for this paper was carried out using the technique of sampling publications for the preparation of scientific reviews [1]. Table 1 contains the protocol for the implementation of this technique.

**Figure 1:**
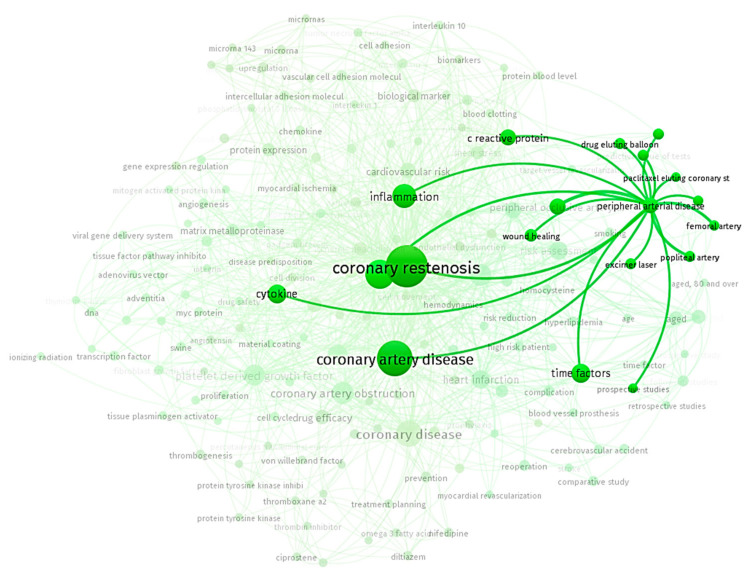
Scientific landscape of restenosis research. The cluster of peripheral arterial decease is emphasized.

Stage 1 searched Scopus for the publications from 2018–2022 containing the term “restenosis” and mentioning “markers” or “risk factors”. At stage 2, on the basis of the sampling results, a network diagram of keywords was built (Figure 1) using the VOS viewer program on the basis of the titles and annotations of articles. A threshold of at least five term occurrences in all papers was applied. After manual inspection, uninformative general usage terms (such as “clinical trial” and “endovascular surgery”) were excluded. Moreover, similar concepts have been identified and combined into one term (e.g., “aged” and “age”). Analysis of the results showed a great amount of research about the coronary vascular disease, but that for the peripheral artery disease was much smaller. Such an uneven distribution indicates a certain imbalance existing in the literature in the studies of restenosis predictors in lower limb arteries and coronary arteries in favor of the latter. We need additional investigations toward findings of common characteristics and differences between peripheral arterial disease (PAD) and coronary restenosis. This review had the aim of attracting attention to this problem.

To achieve the goal of the review, it was necessary to select publications containing information on cases of restenosis against the background of critical limb ischemia. Therefore, at stage 3, the sample was narrowed by adding the following keywords to the search query: “limb ischemia” or “peripheral arteries” or “femoropopliteal arterial”. With the subsequent expansion of the sample with cited documents at stage 4, it included original studies starting from 1990, which are valuable for a comprehensive assessment of the influence of various risk factors on the development of restenosis.

At the fifth stage, irrelevant publications were excluded, namely, those that did not contain information on percutaneous balloon angioplasty with or without stent implantation. At stage 6, the resulting sample was examined by reviewers who left 106 papers on identifying possible predictors of restenosis after the endovascular treatment of patients with atherosclerotic lesions of peripheral arteries. Finally, on the basis of our experience, we added more publications relevant to the subject of the review.

### 1.2. The Role of Atherosclerosis and Diabetes Mellitus in the Development of Restenosis

Currently, diseases of the cardiovascular system are becoming widespread among the population, and timely treatment improves the quality of patients’ life, as well as contributing to the development of favorable long-term clinical results. Among cardiovascular diseases, chronic obliterating lesions of the arteries of lower extremities are distinguished, which, according to various sources, spread among people aged 50 or older, with an estimated incidence of 200 million individuals worldwide [2,3]. In most cases, the cause of damage to the arteries of lower extremities is atherosclerosis [4]. Depending on the level of damage to the arteries, the aorto-iliac, ilio-femoral, femoral-popliteal, and popliteal-tibial segments are distinguished. As a rule, damage to the arteries below the knee is associated with the simultaneous course of atherosclerosis and diabetes mellitus [5,6]. Diabetes mellitus (DM) increases the progression and severity of the disease in patients with concomitant cardiovascular pathologies [7]. The most severe form of peripheral arterial disease (PAD) is critical ischemia characterized by pain at rest, ischemic ulcers, and insufficient limb revascularization, ultimately increasing the risk of limb loss and cardiovascular mortality [7].

The main direction in the treatment of patients with critical limb ischemia (CLI) is percutaneous transluminal balloon angioplasty (PTBA) of the affected part of the vessel. However, the effectiveness of surgical treatment is not absolute, and restenosis in the place of intervention remains one of the important complications [8]. Thus, for example, according to the literature data, restenosis in the stent of the femoropopliteal segment leads to the impaired patency of the vessel in about 50% of cases within 3 years [9]. The mechanism of restenosis can be associated with endothelial damage, neointimal proliferation, and release of pro-inflammatory factors after balloon angioplasty against the background of existing atherosclerosis in the vessel [10]. To inhibit neointimal proliferation after revascularuzation, including in-stent restenosis, drug-coated balloons are designed. Drug-coated balloons for angioplasty with paclitaxel showed lower recurrent in-stent restenosis (ISR) rates and higher freedom from target lesion revascularization (TLR), mainly in patients with femoro-poplitel lesions [11]. The assessment of markers of restenosis after endovascular revascularization with the application of drug-coated devices is also extremely important because drug-eluting technology definitely has an impact on the timeframe and process of vascular restenosis. However, the most modern clinical research mainly concerns restenosis after angioplasty of coronary arteries or superficial femoral artery and in-stent restenosis, as well as a number of experimental studies of the recent past are also devoted to impact of some medicines to proliferation in arteries of the same localization [12,13,14,15], without mention about arteries below knee.

The presence of diabetes mellitus in a patient worsens the course of restenosis.

According to a number of studies, diabetes mellitus (DM) is one of the main risk factors in patients with peripheral arterial disease [16,17]. Diabetes mellitus leads to the endothelium dysfunction, increases the proliferation of smooth muscle cells, and is also involved in platelet activation [18]. Patients with lower limb artery disease and concomitant diabetes mellitus have a high risk of limb amputation, which is 10 times higher than in patients without DM [19,20]. According to a number of studies, the PAD severity correlates with the duration and severity of hyperglycemia [21]. Studies aimed at assessing the level of glycated hemoglobin (HbA1c) showed its correlation with an increased risk of major adverse cardiovascular events (MACE) [22,23], whereas intensive glycemic control (HbA1c target < 6.0%) was associated with reduced risk of lower-extremity amputation (LEA) and revascularization [24]. Two other single-center studies showed that any inadequate glycemic control leads to adverse clinical outcomes in patients with critical lower limb ischemia [25,26].

Many studies prove that the endovascular interventions (plain balloon angioplasty, drug-coated balloon (DCB) angioplasty, stent placement (bare-metal stent, drug-eluting stent (DES), or covered stent), and atherectomy) may all be reasonable options in specific circumstances and lesion anatomies by infrainguinal leasions [5]. Endovascular revascularization permits the minimal resectional interventions on the foot, stops the ischemia, and minimizes the number of repeated necrectomies and high amputations. The diabetic foot syndrome is represented as a multidisciplinary problem [27]. The combined developed technology, including the surgical treatment of chronic lower limb ischemia in diabetic foot syndrome, can be recommended for implementation as part of additional randomized clinical trials in patients with limb-threatening ischemia and diabetic foot syndrome [28].

### 1.3. General Mechanism of Restenosis after Endovascular Revascularization

Endovascular revascularization leads to damage to the endothelium, triggers repair processes and neointima formation, activates a cascade of inflammatory reactions, and leads to the release of inflammatory factors into the blood [29,30]. Undoubtedly, neointimal hyperplasia after bypass surgery also is critical for local restenosis and prognosis for the patient. The aim of our review was the assessment of restenosis after endovascular revascularization. It is connected with some features of this approach, particularly sufficient length of the lesion and the area of transluminal influence. It especially concerns infra-popliteal lesions. A large percentage of CLI patients have infrapopliteal arterial occlusive disease (IPOD), especially within the diabetic population, where PAD is 3–4 times more common. CLI related to diabetes is often more extensive with multi-level long segmental arterial disease resulting in a 5–30-fold increased rate of amputation [31]. It should be noted that a patient with a history of diabetes mellitus, particularly type 2 diabetes (T2D), contributes to the development of delayed chronic inflammation [32]. The restenosis depends on multiple factors and is a complex process that is not completely understood at the molecular level. To discover the molecular basis of complex pathologies, gene network analysis is now widely used. The authors of this article performed the analysis of a gene network related to vascular restenosis using an automatic tool for the biological network reconstruction and analysis called ANDSystem [33]. The gene network reconstruction and analysis were performed using the ANDSystem tool. It is based on the text-mining technology applied to the scientific publications from PubMed and PubMed Central systems. ANDSystem was developed in the Institute of Cytology and Genetics (Novosibirsk) by Dr. Ivanisenko and colleagues. It has been already successfully used for the analysis of gene networks related to asthma, hypertension, hyperglycemia, diabetic complications, lymphedema, and some other pathologies [33].

**Figure 2:**
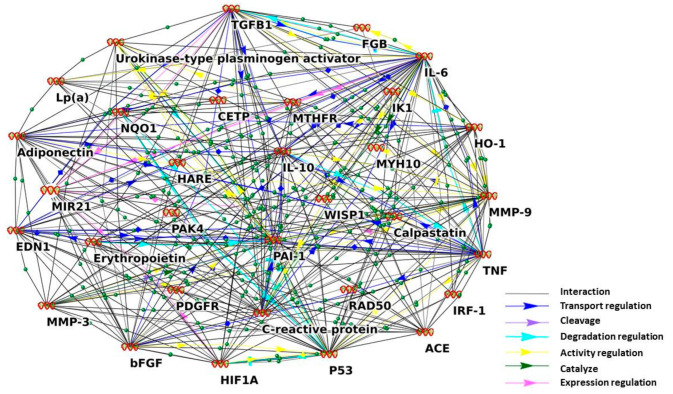
Gene network related to vascular restenosis.

Thus, according to the ANDSystem, we discovered 32 genes that participate in the gene network related to vascular restenosis. In Figure 2, one can see the visualization of this restenosis gene network made by the ANDSystem. Among these 32 genes revealed by the ANDSystem, as related to restenosis, were cytokines, hormones, enzymes, transcription regulators, and some others. To determine the main regulators of this gene network, the number of connections of the genes in the network was analyzed. The genes with the largest numbers of connections with other genes were found in the network. Such genes are often called hubs. These are listed in Figure 2, (IL6, TNF, TP53, SERPINE1, TGFB1, IL10, MMP9, ADIPOQ, CRP, and FGF2). All the 32 genes associated with the restenosis could be divided into three groups. In the first group were plasma proteins, which are markers of restenosis. In the second group were targets for therapeutic strategies against restenosis. Finally, in the third group were other restenosis risk markers [33].

The inflammatory process in the vascular wall through endothelial progenitor cells leads to the proliferation, migration, and differentiation of vascular smooth muscle cells (VSMCs), to the migration of matrix metalloproteinases (MMPs), DNA replication, and the synthesis of the extracellular matrix [30,34]. Molecules of the extracellular matrix are excreted by the smooth muscle cells of the neointima. The extracellular matrix is composed of collagen (type I and III), elastin, glycoproteins, and proteoglycans. Proteoglycans and hyaluronan are involved in the regulation of vascular permeability, lipid metabolism, and thrombosis. In the muscular artery, type III collagen is the most abundant matrix protein [35].

Modeling an artificial arterial injury showed that the neointima formation occurs as a result of the replacement of type III collagen by the type I collagen [36]. In this case, the accumulation of extracellular matrix accumulates mainly around the stent and in the outer intima layer. Moreover, the outer intima layer is characterized by a lower density and lower cell replication compared to the inner intima layer [36]. In a number of experimental and clinical studies, the inflammatory response in response to the injury during balloon angioplasty and/or stenting is considered one of the factors influencing the development of early restenosis [37,38]. These processes form the basis of neointimal hyperplasia. Further, low-density lipoprotein oxidation occurs, as well as activation and migration of macrophages saturated with lipids into the subintimal layer of the vessel, thus forming the necrotic core of atherosclerotic plaque, followed by calcification [39,40,41]. In the vessel intima, certain plasma lipoproteins accumulate, and then they are oxidized and modified. This modification causes ECs to start expressing adhesion molecules (P-selectin, E-selectin, VACM1, and ICAM1) and chemotaxis molecules (CCR2 and CCR5) for monocytes. Monocytes bind to endothelial adhesion molecules and enter the intima, where they differentiate into macrophages in response to locally produced M-CSF and other cytokines. Macrophages engulf modified lipids, becoming foam cells [42]. This process, leading to narrowing the vessel lumen, is called neoatherosclerosis [43]. As is known, monocytes/macrophages are involved in atherosclerosis but also restenosis and were found at hypoxic and sites of the lesion [44]. It is believed that continued investigations of the mechanisms that regulate—and are regulated by—hypoxia-inducible factors (HIF) in specific organs, tissues, and disease states will facilitate the development and refinement of treatments for a wide variety of cardiovascular disorders [45]. Due to this, discussing HIF pathways as one of the fundamental pathogenetic mechanisms is very important. Little is known about the role of the myeloid PHD2 in atherosclerosis and neointima formation. Results of the latest experimental research in vitro showed a distinct expression pattern in differentially polarized macrophages with high expression of Hif-1α, VEGF, and MMP-1 in proinflammatory M1 macrophages. In conclusion, the results show that myeloid Hif-1α is involved in neointima hyperplasia [44].

As shown earlier, ApoA-I supports increased cell proliferation and protects cells from apoptosis in the conditions of oxidative stress and serum deprivation, thus enlarging the viable cell pool [39]. Apolipoprotein A-I/high-density lipoprotein (apoA-I/HDL) levels in T2D patients is reduced. In turn, this leads to a multitude of vascular complications, including coronary artery disease, myocardial infarctions, cerebrovascular disease, peripheral arterial disease, and atherosclerosis [46].

It should be noted that the pathogenesis of atherosclerosis and neoatherosclerosis in the arteries has both common developmental features and differences. Although the focal calcification of the necrotic core in atherosclerotic plaques is present in both processes [47,48], the development of atherosclerosis, in contrast to neoatherosclerosis, can take a long time to develop [49]. The proliferation of VSMCs without infiltration of the site of damage to the vessel by macrophages, as well as calcification of the necrotic nucleus of atherosclerotic plaque, are not mandatory and are absent in the places of arterial stenting and in the development of neoatherosclerosis, respectively [49]. In neoatherosclerosis, macrophages are concentrated more superficially than in atherosclerosis [47]. Moreover, one of the features of neoatherosclerosis is the presence of foam cells, hemorrhages under the atherosclerotic plaque, and the presence of a thin fibrous cap [50,51].

The pathogenesis of neoatherosclerosis may differ not only from that of atherosclerosis but also from the type of stent used during endovascular revascularization. Thus, differences are observed between groups of patients, depending on the presence or absence of drug coating on the stent. The development of neoatherosclerosis in the group of patients using a drug-eluting stent was observed in more cases, and the rate of development was significantly faster, compared to the group of patients where the stent was used without coating [52]. Early neointima in patients treated with a coated stent consisted of fibrin, a small amount of VSMC, and endothelial cells [53]. In the group of patients, where an uncoated stent was used, the early neointima consisted mainly of VSMCs and endothelial cells [40].

A number of pathological conditions that complicate the course of diabetes mellitus contribute to the development and progression of peripheral arterial diseases [19,54]. Mechanisms based on hyperglycemia, dyslipidemia, and insulin resistance trigger the processes by which the vascular wall integrity is violated, endothelial dysfunction occurs, and VSMC proliferation increases; pro-inflammatory factors are released into the blood; the platelet system and the coagulation system are activated, and blood rheology changes [19,55,56] (Table 2).

## 2. Key Pathogenetic Mechanisms of Restenosis Development

### 2.1. The Role of Inflammation in the Development of Restenosis

Conducting endovascular revascularization of the vessel, especially when using a stent, damages the vessel and leads to a local inflammation [72]. A vascular injury induces the secretion of mediators, such as interleukin (IL-1β) and tumor necrosis factor (TNF-α) [73]. Neutrophils, Th1, Th2 lymphocytes, monocytes, platelets, and fibrin are deposited at the sites of injury [73,74,75]. Activated platelets facilitate the attachment of leukocytes to the damaged surface [73]. Moreover, pro-inflammatory processes induce the immune system cells. Tregs play a special role in this process. Treg cells produce anti-inflammatory cytokines, including TGF-β and IL-10 [72,74,75,76]. The study of pro- and anti-inflammatory cytokines is an urgent task in terms of the search for restenosis markers.

Thus, in a study conducted by Guimaraes T. S. et al., the relation was evaluated between the development of early restenosis covered stents and pro- and anti-inflammatory cytokines after stenting the femoropopliteal segment within 6 months after surgery [77]. The study showed a significant increase in the anti-inflammatory cytokines TGF-β and IL-10 and a decrease in the pro-inflammatory cytokines 6 months after the procedure [77]. At the same time, there was no correlation between the pro-inflammatory cytokines and the development of restenosis [77]. Similar results with an absence of correlation between pro-inflammatory cytokines and the development of restenosis were obtained by Araújo, P. V et al. [78]. However, IL-8 levels showed statistically significant reduction 24 h after versus pretreatment (*p* < 0.05), 6 months vs. pretreatment, and 6 months vs. 24 h [78]. As a result, the tumor necrosis factor (TGF-β) signaling was shown to increase the connective tissue growth factor (CTGF) production, thus contributing to the development of widespread progressive fibrous neointimal hyperplasia [79].

Another study by Signorelli et al. evaluated plasma inflammatory markers, namely, interleukin (IL)-6 and tumor necrosis factor (TNF)-α, in patients with peripheral arterial disease compared with controls [80]. During the study, an increase in interleukin (IL)-6 and tumor necrosis factor (TNF)-α were observed, compared with the control group [74]. This issue was also studied by Barani et al. It was found that inflammatory mediators, such as IL-6 and TNF-α, and the highly sensitive C-reactive protein (CRP) correlated with the mortality of patients with critical lower limb ischemia during the year [81]. Di X. et al. analyzed systematic reviews and meta-analyses and confirmed that patients with higher preoperative CRP levels had a higher risk of adverse postoperative events compared with those who had lower baseline CRP levels [82]. Bleda S. and her team found that CRP values > 9.8 mg/L indicate an increased risk of reoperations, which are often open surgical procedures [83]. However, there are no data that would allow us to adjust the tactics of treating the patient in terms of open surgery or endovascular revascularization, since this marker can be a predictor of the patient’s prognosis.

High CRP levels were also associated with serious cardiovascular events in patients with lower limb ischemia [84]. Schillinger’s team of scientists demonstrated that restenesis in patients after percutaneous transluminal angioplasty (PTA) of the distal popliteal and tibioperoneal segment correlated with the elevated levels of CRP and indicated that inflammation plays an important role in the pathophysiological process [85]. Elevated levels of CRP were also found in diabetic patients undergoing lower limb PTA [86]. Bleda et al. analyzed a possible association between CRP levels, fibrinogen levels, and adverse cardiovascular events during 1 year of the study [87]. They found a correlation between basal CRP and fibrinogen levels and reintervention rates, CV events, and death during a follow-up.

In another study conducted by Kotsch et al., the levels of fibrinogen, C-reactive protein (CRP), interleukin 6 (IL-6), interleukin 10 (IL-10), basic fibroblast growth factor (bFGF), and transforming growth factor were determined. The transforming growth factor beta (TGF-b1) was determined in the blood of patients with PAD after endovascular revascularization of the lower extremities and in patients with new onset restenosis. It was found that the concentrations of CRP, fibrinogen, and cytokines (IL-6, bFGF, TGF-b1, and IL-10) were also higher in PAD patients with restenosis, which indicates the involvement of sluggish inflammation in this process [88].

Biscetti et al. hypothesized that there is a correlation between osteoprotegerin (OPG), TNF-α, IL-6, and CRP levels and restenesis in patients with diabetes mellitus, PAD, and critical lower limb ischemia [89]. Indeed, osteoprotegrin plays a role in the calcium metabolism and directly interacts with the vascular endothelium; therefore, it is a marker in patients with atherosclerosis and diabetes mellitus [90,91,92,93]. TNF-α plays a key role in the development of diabetes in atherosclerosis [18]. IL-6 has a positive effect on the endothelial function and aortic stiffness, as evidenced by the data from patients treated with IL-6 inhibitors [94]. CRP promotes the formation of foam cells in atherosclerotic plaque and platelet adhesion [95]. The study found a trend between increased levels of each cytokine and the risk of postoperative complications in patients with diabetes, PAD, and chronic limb-threatening ischemia (CLTI) [89].

In addition to pro-inflammatory cytokines, a significant role is played by nuclear proteins that control gene expression and trigger pro-inflammatory reactions; one of these proteins is highly mobile group box-1 (HMGB-1) [96,97,98]. The relation between HMGB-1 levels, diabetes, and its complications is described in [99]. Oozawa et al. found elevated plasma levels of HMGB1 in patients with diabetes and peripheral arterial disease [100].

### 2.2. The Ratio of Neutrophils to Lymphocytes as a Predictor of Restenosis

One marker of inflammation is the neutrophil to lymphocyte ratio (NLR), which was correlated with increased cardiovascular mortality and morbidity in a number of studies [101,102]. The relation between the ratio of neutrophils to lymphocytes (NLR) with various inflammatory biomarkers was analyzed in a number of studies [103,104,105]. However, there are very few data on the change in NLR before and after a surgical intervention on vessels, in particular on the superficial femoral artery. Yang et al. showed that in patients with restenosis within 12 months, the neutrophil-to-lymphocyte ratio (NLR) increased after stent implantation [106]. A positive correlation was shown between the NLR ratio and the occurrence of restenoses. Thus, postoperative patients with an NLR change > 1.24 had a 2.13 times higher risk of restenosis than patients with an NLR change < 1.24 [106].

The neutrophil-to-lymphocyte ratio (NLR) was proposed as a marker for predicting the patency after the endovascular intervention in the femoropopliteal segment in patients with CLI [107,108]. Restenosis after endovascular intervention is the main complication after surgery. Restenosis rates are high, ranging from 30% to 80% for 1 year [109]. Chan et al. showed that patients with NLR > 5.25 had an increased risk of death compared with those with NLR < 5.25. Thus, the NLR and absolute lymphocyte count are potentially valuable prognostic markers for risk stratification in CLLI patients undergoing popliteal angioplasty [107]. NLR is a marker of the systemic inflammatory process and, if elevated, can lead to more aggressive neointimal hyperplasia and restenosis [107].

### 2.3. Markers of Oxidative Stress

Oxidative stress plays an important role in the development of vascular restenoses. Ganjali S. et al. analyzed the markers of oxidative stress as predictors of restenosis after endovascular revascularization [110]. The study included the following factors: malondialdehyde (MDA), thiol groups (GSH), total antioxidant activity, and activity of serum antioxidant enzymes such as glutathione peroxidase (GPx) and superoxide dismutase (SOD) [110]. Total oxidative load was assessed using a pro-oxidant-antioxidant balance (PAB) analysis. The groups of patients with and without restenosis after PTBA and the control group were compared. The evidence suggests that several oxidative and antioxidant markers, such as total oxidative load and SOD, showed potential in predicting the ISR risk [110].

### 2.4. Hemostasis Factors

The structure of atherosclerotic plaque includes hemostasis components, including tissue factor (TF) and its inhibitors (TFPI) [111,112]. Tissue factor is the main initiator of the extrinsic coagulation pathway and the main factor inducing the fibrin deposition in vivo. According to the Ross theory, the primary event in the development of atherosclerotic plaques in the arterial wall is damage to endothelial and smooth muscle cells by various pathological agents, including high blood pressure, oxidized low-density lipoprotein (LDL), immune or mechanical damaging factors, etc. [113]. After damage to the vascular endothelium, the tissue factor is released, and the receptors for coagulation factor VII are expressed [114]. The TF/VIIa complex is assembled on the cell surface, interacting with the tissue factor inhibitor (TFPI) [114,115]. Thrombomodulin binds to thrombin, forming a thrombomodulin–thrombin complex and activating factors V, VIII, XII, and I, and promotes the conversion of fibrinogen to fibrin and also blocks the TFPI factor [116]. The damaged endothelium secretes von Willebrand factor, which promotes platelet adhesion and interacts with the tissue factor [117,118]. It should be noted that the intact endothelium secretes the tissue plasminogen activator (t-PA), which leads to the plasminogen conversion to plasmin, followed by a fibrin cleavage to D-dimers [119]. Further, the tissue factor/factor VIIa complex triggers an external blood coagulation pathway, which leads to an increase in the thrombin formation and an increase in the concentration of thrombin–antithrombin complexes [114]. A high level of tissue factor and TAT complexes in PAD may indicate a hypercoagulable state and a high risk of thromboembolic complications. As with other forms of atherosclerosis (e.g., cardiovascular disease and PAD), the tissue factor release is a tissue response to a damage to the arterial wall.

Kotschy et al. assessed the level of tissue factor (TF), tissue factor inhibitor (TFPI), thrombin–antithrombin complex (TAT), fibrinogen, and D-dimer in patients with lower limb artery disease after endovascular revascularization [114]. In the group of patients with peripheral arterial diseases after endovascular revascularization, the levels of tissue factor, TAT complex, D-dimers, and fibrinogen were increased, compared to the control group [120]. It was also found that the levels of tissue factor and fibrinogen in patients with restenosis were higher than in the group of patients with PAD before restenosis [120].

Kalinin et al. evaluated the following coagulation factors: VIII, IX, XI, von Willebrand factor, protein C, and plasmiogen activator inhibitor (PAI-1) in patients after bypass surgery and endovascular revascularization, as well as groups of patients with conservative treatment. An increase in von Willebrand factor correlated with shunt thrombosis, myocardial infarction, and restenosis. Plasmiogen activator inhibitor (PAI-1) was elevated in patients who received conservative treatment against the background of PAD progression [121]. An increase in the tissue factor in patients with in-stent restenosis after endovascular revascularization was also confirmed in another study by Kotschy et al. [122].

Thus, it was shown that the levels of fibrinogen, tissue factor, von Willebrand factor, and plasmiogen activator inhibitor (PAI-1) can be predictors of restenosis in patients after endovascular revascularization.

### 2.5. MicroRNA

MicroRNAs are one of the important regulators of restenosis and thrombosis processes after endovascular revascularization. MicroRNAs (miRNAs) are small noncoding regulatory RNA sequences that affect gene expression at the posttranscriptional level by suppressing translation or by degrading miRNA [123]. Experiments performed on in vitro and in vivo models of atherosclerosis revealed the involvement of various miRNAs in endothelial dysfunction, inflammation, angiogenesis, and vascular smooth muscle cell (VSMC) proliferation, thus confirming their role in vascular restenosis [124,125,126,127].

Stojkovic et al. analyzed the role of various microRNAs in the development of atherosclerosis after balloon angioplasty with popliteal stenting. The choice of miRNAs was based on the published data on the current topic [126,127,128]. The study proved that miRNA-195 correlates with the risk of adverse ischemic events in the arteries of lower extremities after endovascular angioplasty and can be an effective predictor of restenosis in the vessels [128].

### 2.6. The Kinin–Kallikrein System

Kinins have both pro-inflammatory and cardioprotective activity and are also involved in various physiological and pathological processes, including blood pressure control, in the cascade of biochemical reactions of coagulation and inflammation [129]. Therefore, the study of the role of the kinin–kallikrein system remains an important task in understanding the mechanisms of restenosis formation.

The kinin–kallikrein system (KKS) is a cascade of biochemical reactions leading to the release of vasoactive kinins. This complex system includes kinin precursors, kininogens, and kallikrein–serine proteases. Kinins are rapidly hydrolyzed by a group of peptidases, kinases. Kininase II, known as the angiotensin-converting enzyme (ACE), is the most studied kininase, and its function is to degrade biologically active kinins, especially bradykinin. Kininase II is also involved in the conversion of angiotensin (Ang) I to Ang II, which plays a role in the endothelial dysfunction [130].

Rocha et al. evaluated the role of kininogen and kinins in the development of restenoses in patients after the balloon angioplasty with stenting in the femoropopliteal segment. It was found that in patients with developed restenosis, the levels of kininogens were significantly lower than in patients without restenosis [131]. However, analysis of plasma and tissue kallikrein, kininase II, and MMP activity levels did not significantly differ between patients with and without restenosis. This study demonstrates the involvement of the kallikrein–kinin system in stent restenosis; however, the involvement of the kinin–kallikrein system needs to be studied in more detail [131].

### 2.7. Metalloproteinases

Matrix metalloproteinases (MMPs) are a family of zinc-dependent endopeptidases widely known for their ability to cleave various protein components of the extracellular matrix and promote its remodeling [132]. Unbalanced MMP activity contributes to the progression of atherosclerosis, participating in processes such as cell migration, SMC proliferation, and infiltration of vessel walls with inflammatory elements [133,134]. The inhibition of MMP activity was proposed as a pharmacological approach to the treatment of cardiovascular diseases [135,136]. According to the literature, MMP-2 and MMP-9 play an important role in various cardiovascular diseases [137].

Metalloproteinase inhibitors (TIMPs) are small proteins, that interact with the zinc-binding active site of the catalytic domain at an equimolar ratio. Four types of TIMP have been described: TIMP-1, TIMP-2, TIMP-3, and TIMP-4. As a rule, TIMP-2 interacts with MMP-2, and TIMP-1 interacts with MMP-9 [132,136]. Currently, it is important to study the role of metalloproteinases and metalloproteinase inhibitors in the development of stenosis after PTBA.

Rocha et al. evaluated the role of MMP-2 and MMP-9, as well as TIMP-1 and TIMP-2, in the development of restenosis. On the basis of the results, the association of restenosis with the level of metalloproteinases or inhibitors of metalloproteinases was not confirmed [131].

### 2.8. The Role of Osteopontin and Osteoprotegrin

Osteoprotegerin (OPG) and osteopontin (OPN) play a role in inhibiting the atherosclerotic plaque calcification in atherosclerosis in patients with PAD and T2D [138]. Osteoprotegrin (OPG) belongs to the glycoproteins of the α-necrosis factor receptor superfamily and is involved in bone resorption [139]. It is produced by various organs and tissues (lungs, intestines, kidneys, bones, cardiovascular system), as well as hematopoietic and immune systems. In the vascular system, early animal studies showed the inhibition of vascular calcification [140,141].

Thus, Kadoglou et al. studied the correlation of osteoprotegerin and osteopontin in the blood serum of patients with peripheral arterial disease with adverse cardiovascular events after endovascular revascularization [142]. The study showed that the level of osteoprotegerin (OPG) and osteopontin (OPN) in patients with PAD compared with the control group corresponded to (OPG) 9.89 ± 2.85 ng/mL vs. 3.47 ± 1.95 ng/mL, and (OPN) 79.99 ± 38.29 ng/mL vs. 35.21 ± 14.84 ng/mL. Moreover, a comparison of the same indicators in groups with and without adverse cardiovascular outcomes was, respectively, (OPG) 13.29 ± 3.23 ng/mL vs. 10.86 ± 3 ng/mL and (OPN) 96.45 ± 40.12 ng/mL vs. 78.1 ± 38.29 ng/mL). Baseline high levels of OPG and OPN were independently associated with the presence of PAD [142].

The correlation between osteopontin and PAD is not well understood; however, the literature data presented support a positive association. One study compared the osteopontin levels in patients with PAD and DM with those in controls. The plasma levels of osteopontin in patients with PAD, compared with controls, were significantly higher than in those without it, regardless of their glycemic status [143].

Although the exact pathophysiological contribution of osteopontin and osteoprotegrin in patients with PAD is still to be elucidated, it can be hypothesized that these parameters may serve as possible biomarkers of restenosis after endovascular revascularization.

## 3. Conclusions

The current data on the pathogenesis and possible predictors of restenosis after the endovascular treatment of patients with atherosclerotic lesions of peripheral arteries are described in the review. This information can be used to further plan actions during the conduct of clinical trials and, subsequently, to develop more accurate updated clinical guidelines for the early diagnosis, prevention, and new approaches to the treatment of critical limb ischemia.

Vascular restenosis associated with neoatherosclerosis remains an important problem in global healthcare. In recent years, systematic reviews and meta-analyses were published and widely disseminated among physicians involved in this topic. However, it should be noted that the mechanisms of restenosis and their possible predictors after balloon angioplasty with stenting are described in more detail in the modern literature, along with studies of restenoses after endovascular revascularization without stenting of peripheral arteries below the inguinal ligament, as well as, in particular, the arteries of the leg being practically absent. There is also a certain imbalance in studies of restenosis in the arteries of the lower extremities and coronary arteries, in favor of the latter. At the same time, some inflammatory markers in these pathologies have common features, but there are undoubtedly differences associated with a greater extent of lesions and the influence of biomechanical parameters on the development of restenosis. Thus, studies aimed at studying the mechanisms of pathogenesis and searching for markers of restenosis of lower limb arteries after balloon angioplasty without stenting will be relevant in predicting the outcomes after a surgical treatment and will also help determine the management features of this group of patients.

## Figures and Tables

**Table 1:** Publication selection protocol.

Stage	Inclusion/Exclusion Criteria	Stage Results
1. Initial search	Scopus query (November, 2022): TITLE-ABS-KEY (restenosis) AND KEY (markers or «risk factors») AND (PUBYEAR AFT 2017) AND (PUBYEAR BEF 2023)	1781 publ.
2. Analysis		The bibliometric analysis revealed an imbalance in covering the problems of predictors of restenosis in the arteries of lower extremities and coronary arteries
3. Record exclusion	Excluded publications, which do not contain the keywords “limb ischemia”, “peripheral arteries”, “femoropopliteal arterial”	48 reviews, 139 articles
4. Sample expansion	All cited documents from the stage 2 sample	8136 publ.
5. Record exclusion	Exclude publications that do not contain “angioplasty” in TITLE-ABS-KEY	651
6. Screening	Examine titles and abstracts by reviewers to exclude articles that do not meet the aims of the paper	13 reviews, 93 articles

**Table 2:** Main links and mechanisms of pathogenesis in restenosis.

Links of Pathogenesis in Restenosis	Basic Mechanisms	Key Markers
Inflammatory process	1. Association of C-reactive protein (CRP) and inflammatory responses with both PAD and DM [56,57]. 2. Promotes the production of procoagulant tissue factor, leukocyte adhesion molecules, and chemoattractants [19]. 3. Inhibits endothelial nitric oxide synthase (eNOS), => reducing nitric oxide (NO) [20,58]. 4. Promotes the production of plasminogen activator inhibitor (PAI)-1, thereby blocking the cleavage of plasminogen into plasmin (impairs the process of fibrinolysis) [59]. 5. An elevated level of (TNF)-α and IL-6 in T2D leads to the binding of cytokines to the receptors on the surface of endothelial cells, the synthesis of adhesion molecules of endothelial cells, which leads to an increase in the binding of leukocytes and platelets to the surface of the endothelium, thereby contributing to thrombosis [60,61,62].	CRP, (eNOS), NO, (TGF-β), IL-6, IL-8, IL-12, (PAI)-1
Endothelial dysfunction	1. Endothelium is involved in the production of endothelin, NO, and reactive oxygen species [63,64]. 2. Hyperglycemia and insulin resistance: indirect effect of insulin on the NO production and relaxation of VSMCs [65,66]. 3. Insulin resistance leads to accumulation of free fatty acids => increase in free oxygen species and decreasing the NO concentration => cannot inhibit the platelet activation and limit migration, and proliferation of VSMC [66,67,68]; 4. Indirect effect on the transformation of macrophages into foam cells, leading to the onset of atherosclerotic plaque formation [20].	Endothelin, NO, reactive oxygen species, free fatty acids
Migration of the VSMC	1. Migration of VSMC from the medial layer to the intimal layer as a stabilization of the atherosclerotic plaque; it is not typical of patients with DM [19,69]. 2. Production of metalloproteinases that destroy collagen, thereby leading to plaque destabilization [70]. 3. Activation of the endothelin-A receptor on the surface of VSMCs, followed by the secretion of endothelin-1, causing pathological vasoconstriction [71].	VSMC, endothelin-1
Platelet dysfunction	1. Activation of protein kinase C and decrease in NO concentration [48]. 2. Increased adhesion of platelets due to the increased expression of P-selectin on the surface of platelets [19]. 3. Increased expression of platelet receptors, such as glycoprotein Ib (which binds to von Willebrand factor) and IIb/IIIa receptors. These receptors mediate platelet adhesion and aggregation, thereby causing thrombosis [19].	Protein kinase C, 3-selectin, glycoprotein Ib, IIb/IIIa receptors.
Coagulation	Activation of factor VIIa and tissue factor, suppression of anticoagulants such as antithrombin III, protein S and protein C [19].	Factor VIIa and tissue factor, suppression of antithrombin III, protein S, and protein C
Blood rheology	Increased fibrinogen production [50].	

## Data Availability

Not applicable.

## Abbreviations

ACE	angiotensin-converting enzyme
APOA	apolipoprotein E is encoded by the APOA gene
bFGF	basic fibroblast growth factor
CLI	critical limb ischemia
CLTI	chronic limb-threatening ischemia
CRP	C-reactive protein
DCB	drug-coated balloon
DES	drug-eluting stent
DM	diabetes mellitus
eNOS	endothelial nitric oxide synthase
GPx	glutathione peroxidase
GSH	thiol groups
HIF	hypoxia-inducible factors
HMGB-1	highly mobile group box-1
IL	interleukin
IPOD	infrapopliteal arterial occlusive disease
ISR	in-stent restenosis
KKS	kinin–kallikrein system
LDL	low-density lipoprotein
LEA	limb extremity amputation
MACE	major adverse cardiovascular events
miRNAs	microRNAs
MDA	malondialdehyde
MMPs	matrix metalloproteinases
NLR	neutrophil to lymphocyte ratio
NO	nitric oxide
OPG	osteoprotegerin
OPN	osteopontin
PAI-1	plasminogen activator inhibitor
PAD	peripheral arterial disease
SOD	superoxide dismutase
TAT	thrombin–antithrombin complex
TF	tissue factor
TFPI	tissue factor pathway inhibitor
TGF-b1	transforming growth factor beta1
TIMPs	metalloproteinase inhibitors
TLR	target lesion revascularization
TNF-α	tumor necrosis factor alpha
T2D	type 2 diabetes
VSMCs	vascular smooth muscle cells
